# Cardiac autonomic dysfunction is associated with hypothalamic damage in patients with childhood-onset craniopharyngioma

**DOI:** 10.1371/journal.pone.0246789

**Published:** 2021-02-16

**Authors:** Hae Woon Jung, Hwa Young Kim, Ji Young Kim, Jung-Eun Cheon, In-One Kim, Seung-Ki Kim, Choong Ho Shin, Sei Won Yang, Young Ah Lee

**Affiliations:** 1 Department of Pediatrics, Kyung Hee University Medical Center, Seoul, Republic of Korea; 2 Department of Pediatrics, Seoul National University Children’s Hospital, Seoul National University College of Medicine, Seoul, Republic of Korea; 3 Department of Radiology, Seoul National University Children’s Hospital, Seoul National University College of Medicine, Seoul, Republic of Korea; 4 Department of Neurosurgery, Seoul National University Children’s Hospital, Seoul National University College of Medicine, Seoul, Republic of Korea; University of Essex, UNITED KINGDOM

## Abstract

**Background:**

Autonomic nervous system dysfunction is implicated in the development of hypothalamic obesity. We investigated the relationship between hypothalamic involvement (HI), central obesity, and cardiac autonomic dysfunction by assessing heart rate variability (HRV) indices in patients with childhood-onset craniopharyngioma.

**Methods:**

A cross-sectional study of 48 patients (28 males, 10–30 years old) with hypothalamic damage after childhood-onset craniopharyngioma was performed. Postoperative HI was graded as mild (n = 19) or extensive (n = 29) on magnetic resonance imaging. Anthropometry, body composition and HRV indices including the standard deviation of all normal R-R intervals (SDNN) and total power (TP) as overall variability markers, root-mean square differences of successive R-R intervals (RMSSD) and high frequency (HF) as parasympathetic modulation markers, and low frequency (LF) as a sympathetic/sympathovagal modulation marker were measured.

**Results:**

Patients with extensive HI had increased means of body mass index, waist circumference, and fat mass than those with mild HI (*P* < 0.05, for all). Centrally obese patients had a lower mean HF, a parasympathetic modulation marker, than centrally non-obese patients (*P* < 0.05). The extensive HI group had lower means of overall variability (SDNN and TP), parasympathetic modulation (HF), and sympathetic/sympathovagal modulation (LF) than the mild HI group (*P* < 0.05, for all). The interaction effect of HI and central obesity on HRV indices was not significant. In models adjusted for age, sex, and family history of cardiometabolic disease, the means of the overall variability indices (*P* < 0.05 for both SDNN and TP) and a sympathetic/sympathovagal modulation index (*P* < 0.05 for LF) were lower with extensive HI, without differences according to central obesity.

**Conclusions:**

The reduced HRV indices with extensive HI suggests that hypothalamic damage may contribute to cardiac autonomic dysfunction, underscoring the importance of minimizing hypothalamic damage in patients with childhood-onset craniopharyngioma.

## Introduction

Hypothalamic obesity can occur in patients with craniopharyngioma, especially those with tumors invading the hypothalamus, and can impact long-term quality of life after childhood-onset craniopharyngioma [1, 2]. Hypothalamic obesity, characterized by hyperleptinemia, hyperinsulinemia, and reduced gut hormone responses [3, 4], can be correlated with the extent of hypothalamic damage. Damage to critical hypothalamic nuclei [5] disturbs control of orexigenic and anorexigenic pathways, leading to changes in satiety and feeding behaviors.

Autonomic nervous system (ANS) balance is also controlled by the central nervous system, with the hypothalamus playing a key role in receiving, integrating, and conveying ANS signals for energy homeostasis [6]. ANS dysfunction in patients with craniopharyngioma can contribute to the development of hypothalamic obesity. Reduced sympathetic activity [7, 8] and increased parasympathetic activity [9] have been reported in patients with craniopharyngioma. However, whether the degree of hypothalamic damage is associated with ANS dysfunction in childhood-onset craniopharyngioma has not been thoroughly investigated.

Reduced overall heart rate variability (HRV) and autonomic imbalance are characteristics of ANS dysfunction, which has been associated with the presence of abdominal obesity [10, 11]. Therefore, we aimed to investigate the role of hypothalamic damage and central obesity on cardiac ANS function by evaluating short-term HRV indices such as overall variability, parasympathetic and sympatho/sympathovagal modulation in patients treated for childhood-onset craniopharyngioma.

## Methods

### Subjects

This study included patients with childhood-onset craniopharyngioma who were at least 6 months past initial surgery, with mild or extensive hypothalamic involvement (HI) on postoperative magnetic resonance imaging (MRI). Among a total of 76 patients aged 10–30 years, 6 patients with comorbid diseases (5 patients with type 2 diabetes mellitus and one patient with a tracheostomy), 3 patients taking medications affecting the ANS (ß-blockers, selective serotonin reuptake inhibitors and antiepileptic medication) and 11 patients who chose not to participate, were excluded from the study. As a result, 56 patients underwent cardiac ANS testing between March 2014 and January 2016. After additionally excluding patients without a recent MRI (n = 1), or without evidence of hypothalamic damage on MRI (n = 5) and those with artifacts on cardiac ANS testing (n = 2), 48 patients with mild or extensive hypothalamic damage were included in this study. Written informed consent was obtained from all patients and guardians who participated in the study. The study protocol was approved by the Institutional Review Board of Seoul National University Hospital (IRB No. 1311-079-535).

### Hypothalamic involvement and anthropometric data

A detailed clinical history including information regarding previous operations, medications, and family history was obtained by a retrospective review of the medical records. Two individual pediatric radiologists (Kim, I.O. and Cheon, J.E.), without information on clinical status, performed an objective review and classified the extent of postoperative HI on the most recent MRI according to Puget’s grading system [12]. Patients with negligible hypothalamic damage or residual tumor displacing the hypothalamus were graded 1 and classified as having mild HI, whereas those with extensive hypothalamic damage with an unidentifiable floor of the third ventricle were graded 2 and classified as having extensive HI in our study.

On the day of cardiac ANS testing, height was measured using a Harpenden stadiometer (Holtain Ltd., Crosswell, UK) and weight using a digital scale (150 A; Cas Co. Ltd., Yangju, South Korea). The body mass index (BMI) was calculated accordingly. Height, weight, and BMI were expressed as age- and sex-specific z-scores according to the 2007 Korean national growth charts [13]. Body composition was analyzed by bioimpedance (InBody 770, InBody Co., Seoul, South Korea). Using the threshold for obesity in Asian populations, obesity was defined as a BMI ≥ 95th percentile for children and absolute BMI ≥ 25 kg/m^2^ for adults [14]. Central obesity was defined as a waist circumference (WC) ≥ 90th percentile for age- and sex-specific references in children [13] and as an absolute WC ≥ 90 cm in adult males or ≥ 80 cm in adult females [14].

### Questionnaires

Questionnaires regarding physical activity (International Physical Activity Questionnaire short form; IPAQ-SF) [15] and dietary intake (three-day food diary) were completed. Regular physical activity was assessed according to definitions established by the US Department of Health and Human Services [16]. The three-day food diary was analyzed by a nutritionist by calculating the total caloric intake (Kcal/day).

### Cardiac autonomic function testing

Patients arrived for cardiac ANS testing after avoidance of strenuous physical exercise and caffeinated beverages in the preceding 24 hours. HRV indices were measured using a computer-based system (DiCan, Medicore Co., Seoul, South Korea). Measurements were performed over a five-minute period in the supine position after a 10-minute period of rest and heart rate stabilization. Time domain indices including the standard deviation of all normal R-R intervals (SDNN) and the root-mean square of the difference of successive R-R intervals (RMSSD) were obtained. Frequency domain parameters were analyzed by power spectral analysis of intervals between sequential R waves with resultant calculations of total power (TP), high frequency (HF; 0.15–0.4 Hz), low frequency (LF; 0.04–0.15 Hz), and the LF to HF ratio (LF/HF). The SDNN and TP in their respective time and frequency domains reflect the overall variability. Both RMSSD and HF (in ms^2^ and normalized units, nu) reflect parasympathetic modulation. The physiological interpretation of LF is not as clear, but it may reflect the sympathetic component (especially when expressed in nu) or both sympathetic and parasympathetic components [17].

### Statistical analysis

SPSS for Windows (version 22.0, IBM Corp., Armonk, NY) was used for statistical analyses. Categorical variables were presented by frequency (percentage). Continuous variables were tested for normality by the Kolmogorov-Smirnov test. Continuous variables with normal distributions were described using the mean value (standard deviation, SD) and those with non-normal distributions were described using the median (interquartile range). For analysis of HRV parameters with non-normal distribution, the LF/HF ratio was logarithm transformed whereas the TP, LF (ms^2^) and HF (ms^2^) were square root transformed to approximate normal distributions. The Student *t*-test was used to test the difference in distributions of continuous variables with between two groups. Categorical variables were compared between groups using the chi-squared test. To determine the strength of the linear relationship between continuous and categorical binary variables, the point-biserial correlation coefficient (r_pb_) was calculated and used to estimate the effect size of the difference between groups. The extent of inter-correlations between the HRV indices was analyzed by Pearson’s correlation. Two-way ANOVA was conducted to analyze the effects of the main factors (HI and central obesity) and their interaction effect (HI x central obesity) on each of the HRV variables. Covariates were considered in models using analysis of covariance (ANCOVA), which included a HRV index as the dependent variable, HI (mild = 0, extensive = 1) and central obesity (non-obese = 0, obese = 1) as main factors, and age, sex (male = 0, female = 1), and family history of cardiometabolic disease (no = 0, yes = 1) as adjusting covariates. Since the interaction term (HI x central obesity) was not significant in analysis by two-way ANOVA, the interaction term was not included in the ANCOVA models. Statistical significance was defined as *P* < 0.05.

## Results

### Baseline characteristics

Table 1 shows the clinical characteristics of the patients. The mean age was 18.5 years (SD 4.8, range 10.1–30.8). Initial operations were undertaken between November 1993 and November 2012 at a mean age of 8.0 years (SD 3.8, range 1.7–18.1). The extent of tumor removal included gross total removal (n = 34), near total removal (n = 5), or subtotal removal (n = 9) by open transcranial (n = 42) or transsphenoidal endoscopic (n = 6) approaches. Ten patients received radiation therapy (dose range 50.1–54 Gy) due to limited surgical resection (n = 2) or recurrence (n = 8). The mean postoperative follow-up duration was 10.5 years (SD 5.5, range 1.7–21.1). All patients at the time of final follow up were receiving hormone replacements (hydrocortisone, n = 45; levothyroxine, n = 47; growth hormone, n = 43; and desmopressin, n = 46). Forty-three patients were of pubertal age, of which 41 patients were receiving sex hormone replacement therapy. Obesity was present in 20 (42%) and central obesity was present in 25 (52%) of the patients.

**Table 1:** Baseline characteristics and comparisons according to hypothalamic involvement and central obesity.

	Total (n = 48)	Hypothalamic Involvement		Central Obesity	
		Mild (n = 19)	Extensive (n = 29)	Non-obese (n = 23)	Obese (n = 25)
***Clinical Characteristics***					
Age [years]	18.5 ± 4.8	17.9 ± 4.1	18.9 ± 5.3	16.1 ± 4.2	20.7 ± 4.3ǂ
Male, n (%)	28 (58%)	9 (47%)	19 (66%)	14 (61%)	14 (56%)
Age at initial operation [years]	8.0 ± 3.8	7.1 ± 4.8	8.5 ± 3.0	6.8 ± 3.8	9.1 ± 3.7ǂ
Postoperative years	10.5 ± 5.5	10.8 ± 5.5	10.4 ± 5.6	9.3 ± 5.6	11.7 ± 5.2
Radiation therapy history, n (%)	10 (21%)	5 (26%)	5 (17%)	4 (17%)	6 (24%)
Recurrence history, n (%)	15 (31%)	6 (32%)	9 (31%)	6 (26%)	9 (36%)
Family history of cardiometabolic disease, n (%)	14 (29%)	2 (11%)	12 (41%)*	4 (17%)	10 (40%)
Visual defect, n (%)	29 (61%)	12 (63%)	17 (59%)	14 (61%)	15 (60%)
***Measures of physical activity and dietary intake***					
Regular physical activity, n (%)	9 (19%)	2 (11%)	7 (24%)	3 (13%)	6 (24%)
Total caloric intake [kcal]	1768 (1462–2054)	1744 (1443–1946)	1810 (1486–2115)	1729 (1473–1977)	1810 (1420–2091)
***Anthropometry and obesity measures***					
Height z-score	0.13 ± 1.34	-0.25 ± 1.49	0.38 ± 1.20	-0.42 ± 1.26	0.64 ± 1.23ǂ
BMI z-score	0.95 ± 1.37	0.32 ± 1.41	1.37 ± 1.20*	0.08 ± 1.06	1.75 ± 1.13ǂ
Obesity, n (%)	20 (42%)	6 (32%)	14 (48%)	3 (13%)	17 (85%)ǂ
Fat mass [%]	30.9 ± 6.8	28.3 ± 7.0	32.6 ± 6.3*	26.9 ± 5.8	34.5 ± 5.7ǂ
Waist circumference [cm]^a^	86.5 (77.5–95.4)	82.0 (72.0–92.0)	88.0 (80.5–103.5)*	77.5 (71.5–86.0)	93.4 (87.3–103.3) ǂ
Central obesity, n (%)	25 (52%)	8 (42%)	17 (59%)		

Data expressed as mean ± standard deviation or median (interquartile range)

* *P* < 0.05, for mild vs. extensive HI

^ǂ^ *P* < 0.05 for centrally non-obese vs. obese

^a^ Logarithm transformed data

Abbreviations: BMI, body mass index.

### Clinical characteristics according to hypothalamic involvement and the presence of central obesity

According to the extent of postoperative HI on MRI, patients were categorized into mild HI (n = 19, 40%) and extensive HI (n = 29, 60%) groups. The means of the BMI z-scores (1.37 vs. 0.32 kg/m^2^, *P* = 0.008), WC (88.0 vs. 82.0 cm, *P* = 0.040), and fat mass (32.6 vs. 28.3%, *P* = 0.031) were significantly higher in the extensive HI group than in the mild HI group. However, there were no significant differences in caloric intake and physical activity between the two groups. According to WC based criteria, the patients were categorized into centrally non-obese (n = 23, 48%) and centrally obese (n = 25, 52%) groups. There were significant differences in the means of the age at the time of testing and at the time of initial operation, with the mean ages at both time points being higher in the centrally obese group compared to the centrally non-obese group (Table 1).

### Heart rate variability indices according to hypothalamic involvement and the presence of central obesity

The means of SDNN and TP, both indices of overall variability, were significantly different between the HI groups. The means of both indices were lower in the extensive HI group, compared to the means of the mild HI group (33.1 vs. 46.3 ms, *P* = 0.014 for SDNN; 628 vs. 1736 ms^2^, *P* = 0.004 for TP). Parasympathetic modulation, as demonstrated by RMSSD and HF also showed mean values that were significantly decreased in the extensive HI group (29.5 vs. 41.5 ms, *P* = 0.035 for RMSSD; 221 vs. 345 ms^2^, *P* = 0.031 for HF). In addition, LF, a parameter of sympathetic or sympathovagal modulation, was significantly different between the HI groups, with lower mean LF in the extensive HI group than in the mild HI group (149 vs. 378 ms^2^, *P* = 0.045). The indices with significant differences in means had large effect sizes (>0.5).

When indices of HRV were compared according to the presence of central obesity, HF (ms^2^), an index of parasympathetic modulation, differed significantly between those with and without central obesity. The mean HF (ms^2^) was lower in the centrally obese group, than in the centrally non-obese group (187 vs. 397 ms^2^, *P* = 0.038). There were no significant differences in other HRV indices between the centrally non-obese and obese groups. When HRV indices were compared using BMI-based criteria, there were no significant differences in HRV indices between BMI-based non-obese and obese groups. The associations between fat mass percentage and HRV indices were not significant.

**Fig 1:**
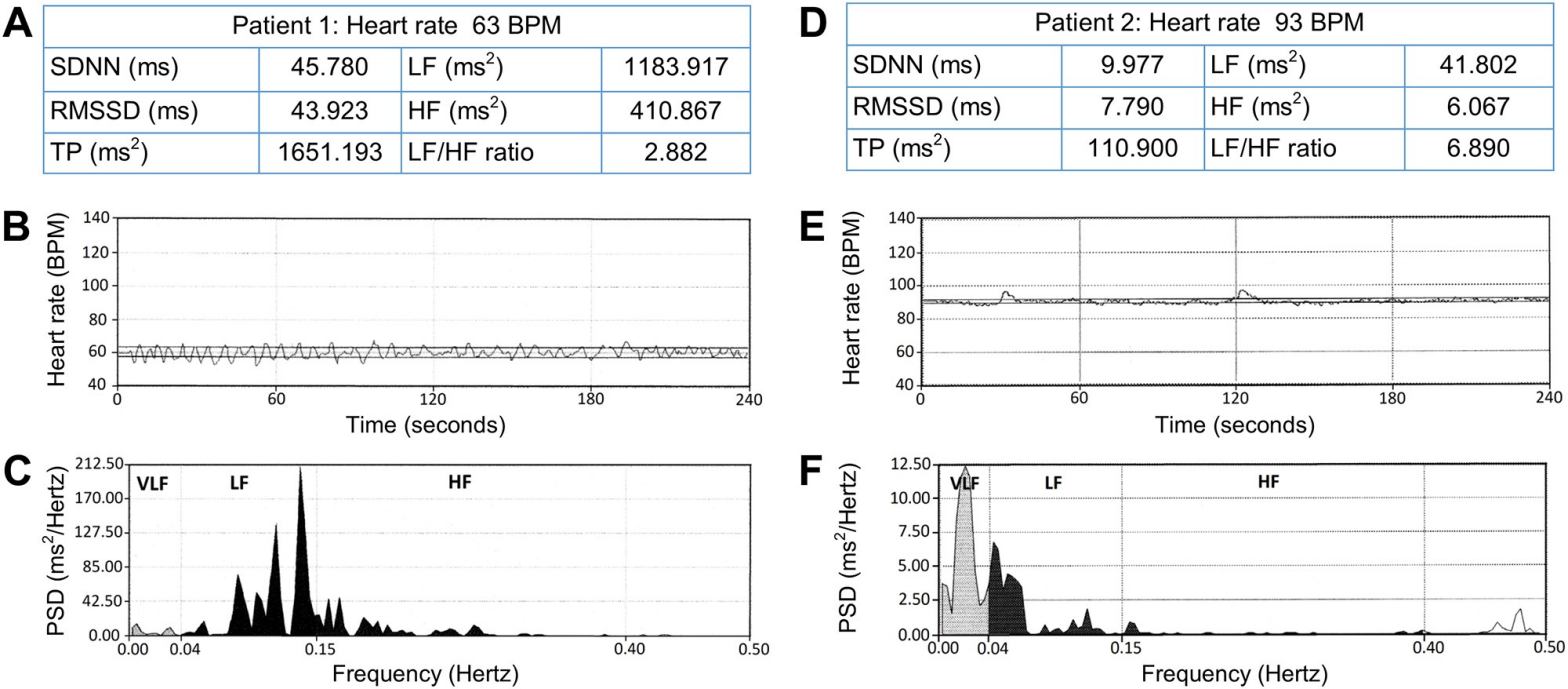
Heart rate variability recordings. Differences in heart rate variability indices (A vs. D), tachograms (B vs. E) and power spectral densities showing distributions of high frequency and low frequency power (C vs. F). Compared to the centrally non-obese Patient 1 (A, B, and C) with mild hypothalamic involvement, heart rate variability indices were visibly reduced in the centrally obese Patient 2 (D, E, and F) with extensive hypothalamic involvement. Note the different scales at the y-axes of the power spectral densities. Abbreviations: BPM, beats per minute; SDNN, standard deviation of all normal R-R intervals; RMSSD, root mean square of the difference of successive R-R intervals; TP, total power; LF, low frequency; HF, high frequency; ms, milliseconds; PSD, power spectral density; VLF, very low frequency.

Table 2 summarizes the comparison results of HRV indices including overall variability (SDNN and TP), parasympathetic modulation (RMSSD and HF), and sympathetic or sympathovagal modulation (LF) according to the grade of HI (mild vs. extensive) and central obesity (centrally non-obese vs. obese). Fig 1 shows the representative recordings of HRV in a centrally non-obese patient with mild HI and a centrally obese patient with extensive HI.

**Table 2:** Heart rate variability indices according to hypothalamic involvement and central obesity.

	Total	HI		Effect size ǂ	CO		Effect size ǂ	Two-way ANOVA (F-values)		
		Mild (n = 19)	Extensive (n = 29)		Non-obese (n = 23)	Obese (n = 25)		HI	CO	HI x CO
SDNN [ms]	38.3 ± 18.4	46.3 ± 16.7	33.1 ± 17.8*	0.76	41.9 ± 17.2	35.0 ± 19.1	0.38	5.432*	0.627	0.613
TP [ms^2^]^a^	1047 (380–1989)	1736 (1029–2708)	628 (209–1592)**	0.91	1142 (579–2630)	793 (343–1976)	0.21	8.546**	0.022	0.530
RMSSD[ms]	34.3 ± 19.5	41.5 ± 20.1	29.5 ± 17.9*	0.63	38.9 ± 19.5	30.0 ± 18.9	0.46	3.682*	1.899	0.280
HF [ms^2^]^a^	253 (100–552)	345 (180–706)	221 (37–472)*	0.66	397 (185–663)	187 (59–338)*	0.62	3.785	3.073	0.543
HF [nu]	48.2 ± 18.6	50.2 ± 17.5	47.0 ± 19.4	0.17	50.8 ± 19.9	45.9 ± 17.4	0.26	0.159	1.393	3.061
LF [ms^2^]^a^	220 (94–441)	378 (199–564)	149 (50–322)*	0.59	290 (140–542)	196 (76–325)	0.30	3.287	0.368	0.420
LF [nu]	51.8 ± 18.6	50.0 ± 17.5	53.0 ± 19.4	0.16	49.2 ± 19.9	54.1 ± 17.4	0.26	0.159	1.393	3.061
LF/HF^b^	1.02 (0.66–1.61)	0.92 (0.65–1.62)	1.16 (0.65–2.05)	0.19	0.8 (0.5–1.8)	1.2 (0.7–1.6)	0.25	0.213	1.197	3.169

Data expressed as mean ± standard deviation or median (interquartile range)

^a^ Square root transformed data

^b^ Logarithm transformed data

*P < .05,

**P < .01,

***P < .001

^ǂ^ Effect size calculated from the point biserial correlation coefficient, indicates a small effect (0.1–0.3), a medium effect (0.3–0.5) or a large effect (>0.5)

Abbreviations: HI, hypothalamic involvement; CO, central obesity; ANOVA (analysis of variance); SDNN, standard deviation of all normal R-R intervals; TP, total power; RMSSD, root mean square of the difference of successive R-R intervals; HF, high frequency; LF, low frequency; ms, milliseconds; nu, normalized units.

**Fig 2:**
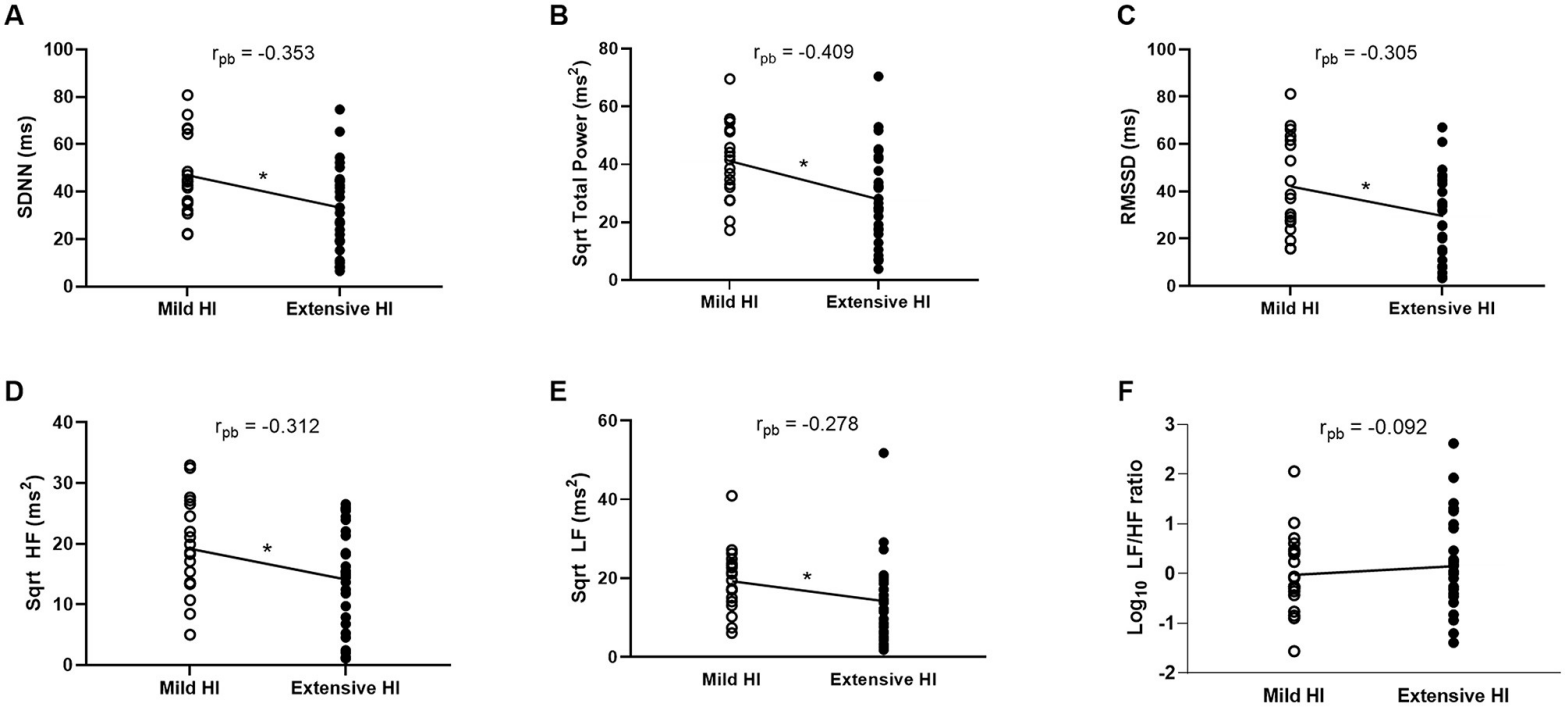
Dot plots of HRV indices and correlations with the grade of hypothalamic involvement. The dot plots of the HRV indices (A) SDNN, (B) TP, (C) RMSSD, (D) HF, (E) LF and (F) LF/HF ratio are compared according to mild (open circles) or extensive (closed dots) hypothalamic involvement groups. The lines show the direction of the correlation with significance marked by asterisks (*P < 0.05). Abbreviations: r_pb_, point-biserial correlation coefficient; SDNN, standard deviation of all normal R-R intervals; RMSSD, root mean square of the difference of successive R-R intervals; HF, high frequency; LF, low frequency; ms, milliseconds; Sqrt, square root transformed.

When point-biserial correlations were conducted, the correlations between the grade of HI and the SDNN (r_pb_ = -0.353), TP (r_pb_ = -0.409), RMSSD (r_pb_ = -0.305), HF (ms^2^) (r_pb_ = -0.312) and LF (ms^2^) (r_pb_ = -0.278) were significant (*P* < 0.05 for all). The dot plots and the direction of the correlations are shown in Fig 2. There were significant differences in the means of SDNN, TP, RMSSD, HF (ms^2^) and LF (ms^2^) between the HI groups, with lower means of HRV indices in the extensive HI group than in the mild HI group. The variability of the HRV indices that could be accounted for by the grade of HI were 12.5% for SDNN, 16.7% for TP, 9.3% for RMSSD, 9.7% for HF (ms^2^) and 7.7% for LF (ms^2^). The different HRV indices are inter-correlated and their correlation coefficients are shown in a (S1 Table).

A two-way ANOVA was conducted to examine the relationship of the two main factors (HI and central obesity) and their interaction term (HI x central obesity) with the HRV indices (Table 2). The interaction term (HI x central obesity) was not significant for all HRV indices. The main effect of HI on indices of overall variability (SDNN and TP) was statistically significant (F_1,44_ = 5.432, *P* = 0.022 for SDNN and F_1,44_ = 8.546, *P* = 0.005 for TP). Extensive HI was associated with a mean SDNN 12.4 ms (95%CI, -22.9 to -1.9) and a mean TP 13.5 ms^2^ (95%CI, -22.9 to -4.2) lower than mild HI. However, the main effect of central obesity on all HRV indices was not significant.

### Is extensive hypothalamic involvement associated with lower HRV?

ANCOVA was conducted to determine the effect of HI and central obesity on HRV indices after controlling for sex, age, and family history of cardiometabolic diseases. There were statistically significant differences in SDNN, TP, and LF (ms^2^) between the mild and extensive HI groups, after adjustment for covariates, while no statistically significant differences in any of the HRV indices were found between centrally non-obese and obese groups. The results are shown in Table 3. The adjusted means of SDNN and TP, as markers of overall variability, were significantly lower in the extensive HI group than in the mild HI group, with an adjusted mean difference of -11.68 ms^2^ (95% CI, -23.01 to -0.31, *P* = 0.044) for SDNN and -14.20 ms^2^ (95% CI, -24.32 to -4.08, *P* = 0.007) for TP. There was also a statistically significant difference in LF (ms^2^), a marker of sympathetic or sympathovagal modulation between the mild and extensive HI groups, after adjustment. The adjusted mean LF was significantly lower in the extensive HI group, with an adjusted mean difference of -5.92 (95% CI, -11.81 to -0.03, *P* = 0.049).

**Table 3:** ANCOVA models to investigate the role of hypothalamic involvement and central obesity on heart rate variability indices.

	Dependent variables											
	SDNN (ms)		TP (ms^2^)^a^		RMSSD (ms)		LF (ms^2^)^a^		HF (ms^2^)^a^		LF/HF ^b^	
Independent variables	MD (95%CI)	*P*	MD (95%CI)	*P*	MD (95%CI)	*P*	MD (95%CI)	*P*	MD (95%CI)	*P*	MD (95%CI)	*P*
Extensive HI (vs. mild HI)	-11.7 (-23.0, -0.3)	0.044	-14.2 (-24.3, -4.1)	0.007	-8.7 (-20.7, 3.4)	0.153	-5.9 (-11.8, -0.03)	0.049	-3.7 (-8.9, 1.4)	0.150	-0.1 (-0.7, 0.4)	0.578
Centrally obese (vs. non-obese)	-2.0 (-14.5, 10.5)	0.748	0.1 (-11.0, 11.2)	0.985	-4.5 (-17.7, 8.7)	0.491	0.4 (-6.1, 6.8)	0.907	-2.9 (-8.6, 2.7)	0.299	0.2 (-0.4, 0.7)	0.565
Age (per 1yr)	-0.5 (-1.8, 0.8)	0.417	-0.3 (-1.4, 0.8)	0.592	-0.4 (-1.8, 0.9)	0.526	-0.6 (-1.2, 0.1)	0.082	-0.2 (-0.8, 0.4)	0.500	-0.01 (-0.07, 0.05)	0.689
Male (vs. female)	1.5 (-9.9, 12.8)	0.796	3.0 (-7.1, 13.1)	0.552	-3.5 (-15.5, 8.6)	0.565	2.3 (-3.6, 8.2)	0.442	-2.8 (-8.0, 2.4)	0.278	0.5 (-0.1, 1.0)	0.056
Family history ^c^ (vs. no history)	-2.8 (-15.5, 10.0)	0.664	0.8 (-10.6, 12.1)	0.892	-5.0 (-18.5, 8.5)	0.460	2.1 (-4.5, 8.7)	0.523	-1.5 (-7.3, 4.3)	0.600	0.7 (0.1, 1.2)	0.029
R^2^	0.160		0.181		0.163		0.168		0.203		0.235	

Abbreviations: HI, hypothalamic involvement; SDNN, standard deviation of all normal R-R intervals; TP, total power; RMSSD, root mean square of the difference of successive R-R intervals; HF, high frequency; LF, low frequency; ms, milliseconds; MD, mean difference; CI, confidence interval)

^a^ Adjusted mean difference and p- values for square root transformed data

^b^ Adjusted mean difference and p-values for logarithm transformed data

^c^ Family history of cardiometabolic disease.

## Discussion

In patients with hypothalamic damage treated for childhood-onset craniopharyngioma, higher grade hypothalamic damage was associated with increased fat mass, BMI, and central obesity, without differences in dietary intake and physical activity. Patients with extensive HI demonstrated lower means of overall variability (SDNN and TP), parasympathetic modulation (HF), and sympatho/sympathovagal modulation (LF) markers than those with mild HI. Centrally obese patients showed a lower mean parasympathetic modulation index (HF) than the centrally non-obese. The interaction of HI and central obesity on the HRV indices was not significant. After adjusting for age, sex, and family history of cardiometabolic disease as covariates, the overall variability and sympathetic/sympathovagal modulation significantly decreased with higher grade hypothalamic damage, but the HRV indices did not differ according to the presence of central obesity.

More extensive HI with an unidentifiable floor of the third ventricle was associated with greater postoperative BMI z-scores, fat mass, and central obesity in this study. This is consistent with the results of previous studies reporting positive correlations between the extent of HI quantified on MRI and postoperative weight gain [12, 18–20]. When semi-quantitative analysis to assess hypothalamic damage was performed using lesion scores on MRI, lesions of the dorsal hypothalamic area (DHA) and dorsomedial nucleus (DMN) in the posterior hypothalamus were associated with increased risk for rapid and pathological weight gain in the first year following surgery [21]. Generally, structural damage to several medial and posterior hypothalamic nuclei related to satiety signaling pathways causes disruption of orexigenic and anorexigenic feeding circuits, leading to lack of satiety, food seeking behavior, energy overload, and development of hypothalamic obesity [22, 23]. However, there were no significant differences in 3-day dietary intake and physical activity between the mild and extensive HI groups in our study. Other pathophysiologic mechanisms including central leptin and insulin resistance with decreased response to peripheral satiety signals, and dysfunction of central hypothalamic control of the efferent ANS may contribute to hypothalamic obesity [4].

ANS dysregulation has been implicated as one cause of hypothalamic obesity in several human and animal studies. Hypothalamic damage in patients with craniopharyngioma leads to organic leptin resistance in the afferent arm and ANS dysfunction in the efferent arm, promoting inadequate energy expenditure and excessive energy storage [24]. Previous studies have provided evidence of increased efferent parasympathetic activity and suppressed sympathetic activity associated with hypothalamic damage [7, 8, 25]. Augmented parasympathetic activity stimulates insulin secretion by pancreatic β-cells [26] that promotes lipogenesis and decreases energy expenditure, which eventually results in hypothalamic obesity [27, 28]. Lustig et al. have reported that a 6-month trial of octreotide was effective in suppressing insulin secretion and stabilizing weight in children with hypothalamic obesity [27]. Decreased sympathetic outflow has been found in craniopharyngioma patients with HI, suggested by decreased catecholamine metabolites (homovanillic acid and vanillylmandelic acid) in morning voided urine, compared to those without HI [7]. We initially hypothesized that there would be increased parasympathetic and decreased sympathetic activity in obese patients with craniopharyngioma. However, contrary to our initial hypothesis, HF (ms^2^), reflecting parasympathetic activity, was rather decreased in the extensive HI group as well as in the centrally obese group. It remains unclear why our findings are discrepant from previous results of parasympathetic predominance and impaired sympatho-adrenal activation in craniopharyngioma patients with hypothalamic obesity. However, our study findings are consistent with those of a recent study [29], which showed a negative correlation between parasympathetic modulation (HF) and the waist-to-height ratio in obese patients with craniopharyngioma, without differences in HRV indices when compared to BMI-matched control subjects with simple obesity. Previous studies of simple obesity have generally shown decreases in parasympathetic activity with sympathetic predominance [30–34]. Changes in LF may be biphasic in simple obesity, as shown by a study in which LF increased in recent onset obesity but decreased with a longer duration of obesity [32]. Our study is limited by its cross-sectional analysis of patients with craniopharyngioma after a mean postoperative follow-up of 10.8 years, which cannot demonstrate dynamic changes in autonomic function. We also cannot preclude that reduced parasympathetic activity may ensue after a long duration of obesity, as in simple obesity. We could neither evaluate for preoperative cardiac ANS function nor evaluate for longitudinal changes in sympathetic and parasympathetic activity. We can postulate that testing earlier on in the course of treatment, when rapid gains in weight are in progress, and testing serially may better elucidate the preoperative and postoperative changes in sympathetic and parasympathetic activity that lead to the development of hypothalamic obesity.

Overall variability was inversely associated with the grade of HI rather than central obesity in our study, and the interaction effect of HI grade and central obesity on overall variability was not significant. Associations between the hypothalamus and the ANS can be explained by the central role of the hypothalamus in controlling efferent sympathetic and parasympathetic branches of the ANS through the locus coeruleus, ventral lateral medulla, and dorsal vagal complex [6]. As such, a direct association between HI and overall variability measures of HRV seems plausible. These nuclei project control of the sympathetic and parasympathetic nervous systems with effects on skeletal muscle, pancreatic insulin secretion, hepatic glucose production, lipolysis in adipose tissue, myocardial contraction and oxygen consumption [6, 24]. A decrease in overall variability was associated with aging, chronic stress, and disorders leading to pathology or inadequate functioning in the various self-regulatory control systems [35]. Hence, reduced HRV has been demonstrated in a wide range of pathologic states such as immune dysfunction, inflammation, diabetes, metabolic syndrome, and cardiovascular disease [36–42]. We recently reported that cardiac autonomic dysfunction, as evidenced by reduced variability, was associated with increased cardiometabolic risk in patients with childhood-onset craniopharyngioma [43]. The long-term effects of reduced variability on cardiometabolic health outcomes in these patients need to be further investigated. In addition to the overall variability indices, the mean of LF (ms^2^) was also decreased with extensive HI in this study. However, the decrease in LF is difficult to interpret, as the LF component does not necessarily reflect only sympathetic modulatory activity and cannot establish, the direction of the sympathovagal imbalance.

Our study had several limitations. First, this study included only a group of patients with craniopharyngioma, without a control group matched for age and obesity, to which the changes in HRV indices could be put into better perspective. However, our study included patients with craniopharyngioma who were of normal weight, overweight, or obese, allowing for various comparisons between the degree of obesity, HI, and cardiac ANS dysfunction within the patient group. Second, the study was of a cross-sectional design. Thus, the pathophysiologic role of dynamic changes in ANS function on the development or progression of obesity could not be elucidated. Third, this study was limited by its small sample size. A larger patient group would allow for semi-quantitative assessments of HI, which would better represent the spectrum of postoperative HI in craniopharyngioma patients and strengthen the finding of an association between hypothalamic damage and cardiac autonomic dysfunction. Since our measurement of HRV indices at rest reflects only in part, cardiac modulatory functions of the ANS, we could not assess the broader aspects of ANS regulation including blood pressure, thermoregulation, respiration, gastrointestinal, and genitourinary functions, which was another limitation of this study. In addition, there were no adjustments for the multiple comparisons in the study. This is the first exploratory study to demonstrate a significant relationship between the grade of hypothalamic damage and cardiac autonomic dysfunction in patients with childhood-onset craniopharyngioma. Since the aims and results of this study are exploratory, further studies are needed to confirm these findings.

In conclusion, the reduced HRV in patients with more extensive HI implicates a possible role for hypothalamic damage in cardiac autonomic dysfunction and suggests the importance of minimizing hypothalamic damage after childhood-onset craniopharyngioma. Further studies are needed to elucidate the role of changes in sympathetic and parasympathetic activities and the direction of autonomic imbalance on the development of hypothalamic obesity and cardiometabolic disorders in patients with childhood-onset craniopharyngioma.

## Supporting information

S1 Table Pearson correlations among heart rate variability indices. (DOCX)
