# Supplementary material for: Cardiac autonomic dysfunction is associated with hypothalamic damage in patients with childhood-onset craniopharyngioma
Source: PLoS One. 2021 Feb 16;16(2):e0246789. doi: 10.1371/journal.pone.0246789 (PMC7886170; doi:10.1371/journal.pone.0246789)
Supplement: S1 Table — (DOCX) [file pone.0246789.s001.docx]

**S1 Table. Pearson correlations among heart rate variability indices.**

| Variables | SDNN (ms) | TP (ms^2^) ^a^ | RMSSD (ms) | HF (ms^2^) ^a^ | LF (ms^2^) ^a^ | LF/HF ^b^ |
| --- | --- | --- | --- | --- | --- | --- |
| SDNN (ms) | 1 |  |  |  |  |  |
| TP (ms^2^) ^a^ | 0.923** | 1 |  |  |  |  |
| RMSSD (ms) | 0.895** | 0.774** | 1 |  |  |  |
| HF (ms^2^) ^a^ | 0.899** | 0.841** | 0.941** | 1 |  |  |
| LF (ms^2^) ^a^ | 0.813** | 0.879** | 0.612** | 0.699** | 1 |  |
| LF/HF ^b^ | -0.289* | -0.178 | -0.519** | -0.547** | 0.091 | 1 |

*P < 0.05, **P < 0.01
^a^ Square root transformed data
^b^ Logarithm transformed data
Abbreviations: ; SDNN, standard deviation of all normal R-R intervals; TP, total power; RMSSD, root mean square of the difference of successive R-R intervals; LF, low frequency; HF, high frequency; ms, milliseconds
